# Response of the Pacific inter-tropical convergence zone to global cooling and initiation of Antarctic glaciation across the Eocene Oligocene Transition

**DOI:** 10.1038/srep30647

**Published:** 2016-08-10

**Authors:** Kiseong Hyeong, Junichiro Kuroda, Inah Seo, Paul A. Wilson

**Affiliations:** 1 Korea Institute of Ocean Science and Technology, Ansan, South Korea; 2 Japan Agency for Marine-Earth Science and Technology, Yokosuka, Japan; 3 School of Earth and Environmental Sciences, Seoul National University, Seoul, South Korea; 4 National Oceanography Center Southampton, University of Southampton, Waterfront Campus, Southampton, SO14 3ZH, UK

## Abstract

Approximately 34 million years ago across the Eocene–Oligocene transition (EOT), Earth’s climate tipped from a largely unglaciated state into one that sustained large ice sheets on Antarctica. Antarctic glaciation is attributed to a threshold response to slow decline in atmospheric CO_2_ but our understanding of the feedback processes triggered and of climate change on the other contents is limited. Here we present new geochemical records of terrigenous dust accumulating on the sea floor across the EOT from a site in the central equatorial Pacific. We report a change in dust chemistry from an Asian affinity to a Central-South American provenance that occurs geologically synchronously with the initiation of stepwise global cooling, glaciation of Antarctica and aridification on the northern continents. We infer that the inter-tropical convergence zone of intense precipitation extended to our site during late Eocene, at least four degrees latitude further south than today, but that it migrated northwards in step with global cooling and initiation of Antarctic glaciation. Our findings point to an atmospheric teleconnection between extratropical cooling and rainfall climate in the tropics and the mid-latitude belt of the westerlies operating across the most pivotal transition in climate state of the Cenozoic Era.

Approximately 34 million years, Myr, ago, across the Eocene–Oligocene transition (EOT), Earth’s climate underwent a shift from a largely unglaciated state to a state sufficiently cool to sustain extensive ice sheets on Antarctica^1^,^2^. The deep-sea oxygen isotope record in benthic foraminiferal calcite from the Pacific Ocean is characterized by a ∼1.5‰^3^,^4^ increase that takes place rapidly in two (∼40 kyr-long) steps^3^. The form of this record is remarkably similar to that simulated^5^ in coupled climate-ice sheet model experiments wherein rapid glaciation is achieved via a threshold response to slow CO_2_ decline by powerful positive ice-sheet feedbacks that are triggered once the descending snowline intersects Antarctic upland plateaus. Stepwise increases in benthic carbon isotope and carbonate compensation depth (CCD) records across the EOT point to a tightly coupled major carbon cycle perturbation^3^. Geochemical records show evidence of CO_2_ decline^6^,^7^ and cooling globally^8^,^9^,^10^,^11^ but the cryosphere response to cooling was distinctly asymmetric about the equator. Antarctic glaciation was not accompanied by the development of large ice sheets in the northern hemisphere^12^ because the northern continents were positioned at lower latitudes than Antarctica meaning that summers there were warmer for a given level of CO_2_^13^. We know much less about the feedback processes^14^,^15^ triggered by Antarctic glaciation or climate change on the other continents, particularly in their hydroclimate. It is now quite well established that central Asia transitioned rapidly to a more arid climate across the EOT but the cause of this change is the subject of debate and there is deep controversy over the importance of changes in temperature versus precipitation to account for terrestrial records of North American EOT climate^16^,^17^,^18^,^19^,^20^.

To investigate linkages between accelerated cooling, the development of large ice sheets on Antarctica and global atmospheric circulation patterns across the EOT we studied the provenance of the inorganic silicate fraction (pelagic clay) of sediments at Integrated Ocean Drilling Program, IODP, Site U1334 (Fig. 1). We selected Site U1334 (7°59.99′N, 131°58.41′W, 4799 m) for three reasons. First, this is the most expanded EOT section from the Pacific Ocean. Second, its chronology is tied, by detailed cyclostratigraphic correlation^21^, to nearby Ocean Drilling Program, ODP, Site 1218 where a benchmark benthic stable isotope stratigraphy for the EOT has been developed^22^. Third, tectonic reconstructions indicate that, 34 Myr ago, Site U1334 was located close to the palaeo-equator in the central East Pacific (palaeolatitude ∼0.25 ±1 °N)^23^, just south of the main present day influence of the inter-tropical convergence zone, ITCZ, the low latitude low pressure and high rainfall belt formed by the confluence of the northern and southern trade winds marking the axis of the global atmospheric circulation system (Fig. 1a). The significance of this location lies in ITCZ-control of the provenance of the terrigenous aeolian dust accumulating on the sea floor in the Pacific Ocean (Fig. 1b).

The inorganic silicate fraction of the surface sediments accumulating today on the sea floor in the Central Pacific Ocean is a mixture of three primary detrital components with distinct neodymium isotope compositions reflecting continental source areas in Asia, North America, and Central and South America^24^,^25^,^26^,^27^ (Fig. 1b). The influence of North American continental hemipelagic and eolian sources is modest, mostly restricted to the easternmost North Pacific around 30 °N latitude. Today, aeolian dust from Asia is transported to the Pacific Ocean from the arid belt between the north Tibetan Plateau and the Central Asia orogen on the mid-latitude westerlies (Fig. 1a). Some of this dust is returned westwards toward the equatorial region by the northeast trade winds^28^ and dominates deposition over a large area of the central Pacific Ocean west of about 100 degrees longitude and north of about 4 degrees north latitude (the southern margin of the latitudinal band occupied by the ITCZ, Fig. 1). To the south and east of these co-ordinates, deposition is dominated by dust transported by the southeast trades from Central/South America (Fig. 1b). Sharp latitudinal demarcation in the provenance of aeolian dust flux is a consequence of the barrier to cross-equatorial dust transport presented by heavy rainfall associated with the ITCZ that acts to wash dust out of the atmosphere^26^,^29^.

Today the ITCZ shows a marked positional bias to the northern hemisphere in the central to east Pacific Ocean (Fig. 1a) with a distinct cyclic seasonal migration between 4 °N and 10 °N toward the summer hemisphere. Thus, down-core records of pelagic clay chemistry and mineralogy provide a way to assess past behavior of the ITCZ and associated atmospheric circulation. Asian and Central/South American dust are distinctive in both their ^143^Nd/^144^Nd composition (we present data using ε_Nd_ notation, see methods) and in the proportion of light rare earth element (REEs) relative to heavy REEs (presented as Lanthanum (La) to Ytterbium (Yb) ratio)^27^. To track the provenance of aeolian dust accumulating at Site U1334 across the EOT we determined the elemental and radiogenic isotopic composition (ε_Nd_) of the inorganic silicate fraction of sediments.

## Results

Our records show pronounced shifts both in ε_Nd_ and a PAAS (Post-Archean Australian Average Shale^30^)-normalized La to Yb ratio, (La/Yb)_SAMPLE_/(La/Yb)_PAAS_ (hereafter expressed as La/Yb^*^) across the EOT (Fig. 2). The Nd isotope composition of the terrigenous fraction in samples of late Eocene age (>34 Myrs) is distinctly less radiogenic (2.2 ε_Nd_ unit in average) than samples of early Oligocene age. The shift is highly significant compared to analytical error (±0.4 ε_Nd_ unit, 2SD) and standard deviation of ε_Nd_ variation pre (±0.6 ε_Nd_ unit) and post-EOT shift (±0.5 ε_Nd_ unit). The transition to more radiogenic values takes place rapidly (∼250 kyr) during the latest Eocene and it is associated with a shift in La/Yb* from LREE (light rare earth element)-enriched to -depleted ratios (Fig. 2).

## Discussion

**Figure 1:**
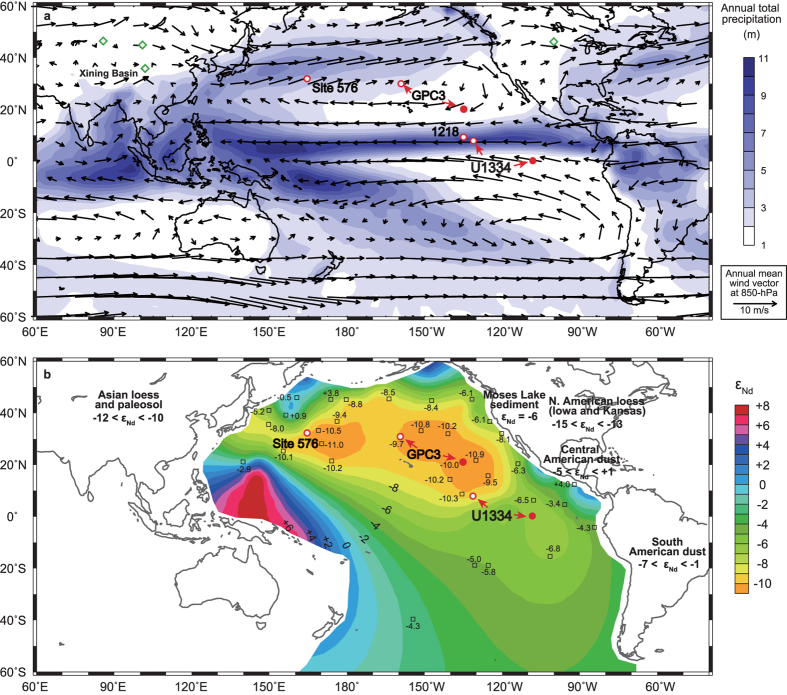
Precipitation, wind fields, and contoured ε_Nd_. (**a**) Annual total precipitation (contours)^57^ and annual average wind fields at 850-hPa (vectors)^58^ and (**b**) contoured neodymium (Nd) isotopic ratio (ε_Nd_) of core-top sea-floor sediments and ε_Nd_ signatures of potential dust sources, see refs 24,27,38,59, 60, 61, 62 for data sources. Palaeo (34 Ma) positions of Site U1334 and LL44-GPC3 at 34 Ma are shown as red circles, present locations shown as white circles with that of ODP Site 1218 and DSDP Site 576. The band of heavy low latitude precipitation indicates the ITCZ. Diamonds indicate present day locations of terrestrial EOT sections, Xining Basin^17^, northwestern China^49^, southern Mongolia^48^, and central North America^18^. These figures are generated by Grid Analysis and Display System (GrADS) Version 2.1.

Our data set falls into two distinct groups in a ε_Nd_ − La/Yb^*^ cross plot (Fig. 3). Data from the latest Eocene and early Oligocene in our record have Nd isotope and REE compositions with a modern day Central/South American dust affinity (Fig. 3), the same provenance as dust accumulating at our study site today (Fig. 1a). Data from the late Eocene (≥34 Ma) portion of our record, however, show a very different composition that is consistent with an Asian dust source, albeit slightly more radiogenic than the composition of dust accumulating today in the central North Pacific Ocean (Figs 1b and 3). To assess the cause of this implied change in provenance accumulating at our study site across the EOT we must first assess the extent to which modern dust sources and their geochemical compositions are representative of Paleogene conditions.

Today dust accumulation in the North Pacific Ocean is dominated by flux from Asia transported on the westerlies. Two lines of evidence strongly suggest that the same was true of the North Pacific during the Eocene and Oligocene. First, the Asian paleosol and lacustrine δ^18^O record show that the westerlies have acted as the main agent for moisture transport to– and aridity of Central Asia since the mid Eocene^31^. Second, records of mass accumulation rates for aeolian dust in the North Pacific Ocean show a strong west-to-east decrease between approximately 45 and 25 Myrs ago^32^. ε_Nd_ records from the dust deposits of the Chinese Loess Plateau (CLP)^33^ show a similar pattern of change over time to those recorded in these North Pacific sediment archives and these changes are extremely modest and gradual in comparison to the large and rapid down core shift that we document at Site U1334 across the EOT. These simple observations, together with the distinctly radiogenic ε_Nd_ values attained in our record from Site U1334 (Fig. 3), strongly suggest that there is no way to explain our data by invoking change in the composition of the dominant (Asian) dust source to the North Pacific across the EOT.

The Mio-Pliocene origins of the large deserts of North America^34^ and North Africa^35^,^36^ rules out these potential sources as having exerted a significant influence. South American sources of dust to the central Pacific such as the Atacama Desert are, by contrast, ancient but the ε_Nd_ composition of parent Mesozoic to Cenozoic volcanic rocks exposed in the Chilean and Argentinian (Patagonia) regions (−7 to +4, see ref. 37 and references therein) are consistent with those (−7 to −1^38^) of South American dust delivered to the Pacific Ocean today. These observations suggest that the shift in terrigenous composition documented in our records indicates a rapid (∼250 kyr) switch in the source of eolian dust supply to Site U1334 from Asia (late Eocene) to Central/South America (latest Eocene and earliest Oligocene).

Pacific tectonic plate motion is too slow (∼0.27 degrees latitude per Myr) and the switch between the two provenance regimes is of the wrong sign (Asian-to-Central/South American) to be explained by northward transport of our study site from one latitudinal depositional regime to another. Long-term records of aeolian flux^26^ from the core of the present day depocentre of Asian dust in the central North Pacific Ocean (e.g., Site GPC3 and DSDP 576, Fig. 1b), together with records^17^,^39^ of continental aridity for the EOT from the north of Tibet, indicate that our findings cannot be explained by decreased Asian dust supply to the North Pacific Ocean across the EOT. Instead, the provenance switch suggests an atmospheric control involving a change in ITCZ behavior. It is unlikely that ITCZ rainfall became a more effective wash out mechanism for Asian dust across the EOT because of surface ocean cooling^8^,^9^ and therefore decreased water vapour supply to the atmosphere^16^. But our data can be explained by a northward migration in ITCZ meridional range. We suggest that the ITCZ was positioned over our study site during dust transport season (boreal winter-spring) in the late Eocene but then migrated north during the latest Eocene-earliest Oligocene, nearer to its present day position in the central Pacific Ocean where atmospheric washout acts as a barrier to equatorial penetration of Asian dust resulting in a modern day Central/South American provenance at our site.

**Figure 2:**
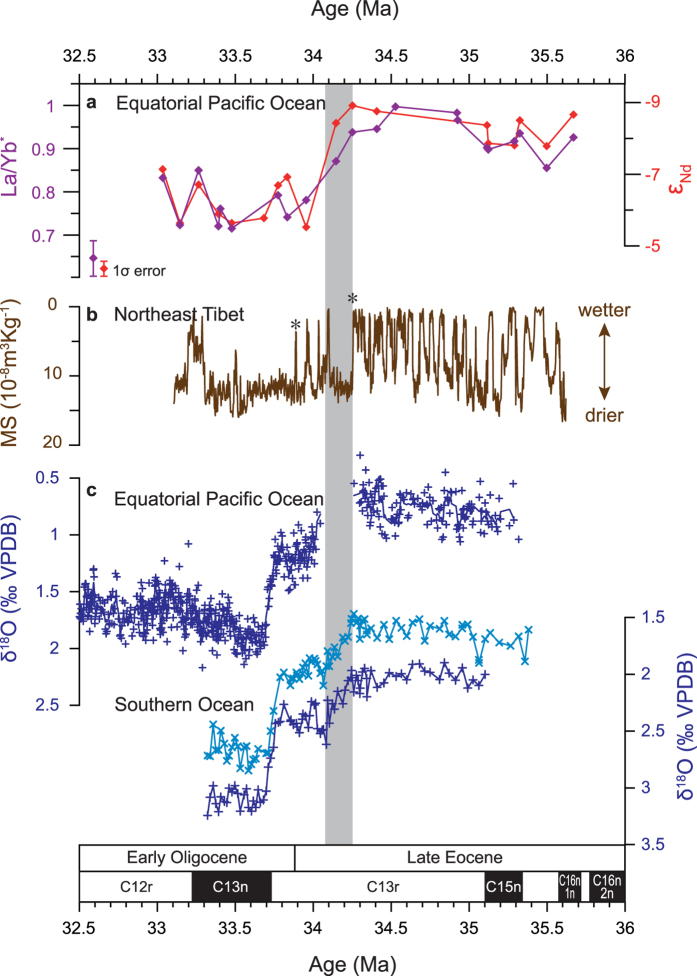
Inter-hemisphere changes in EOT climate. Palaeoclimate records for the Eocene-Oligocene transition from the equatorial Pacific Ocean, Northeast Tibet and the Southern Ocean. (**a**) ε_Nd_ (red) and La/Yb* (purple) of the inorganic silicate fraction of deep sea sediments from IODP U1334 (this study). (**b**) magnetic susceptibility from the Xining Basin, Tashan section (ref. 39). (**c**) oxygen isotope composition of benthic (*Cibicidoides* spp., dark blue) and planktic (*S. angiporoides*, light blue) foraminiferal calcite from ODP Sites 1218 (refs 3,22) and 689 (ref. 10). Magnetic susceptibility (MS) of the Tashan section reflects lithological change in a sequence of red mudstones (high MS) signifying arid conditions and gypsum/gypsiferous layers (low MS) that signify higher water supply. Asterisks in panel (b) show position of two tie points, in addition to magnetostratigraphic datums, used for the correlation of terrestrial to marine records (see methods).

**Figure 3:**
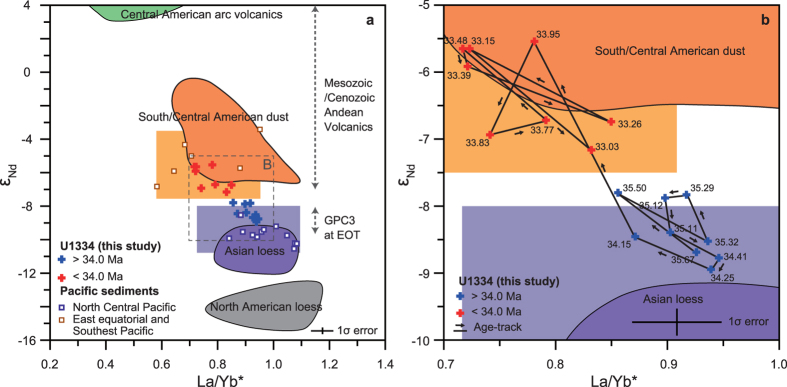
ε_Nd_ vs. La/Yb*. (**a**) ε_Nd_ vs. La/Yb* cross plot for the inorganic silicate fraction of deep sea sediments from IODP Site U1334 (+symbols; blue >34 Myr, red <34 Myr) compared to data from Pacific Ocean sediments and potential continental source regions. Orange signifies the South/Central American dust source (dark shaded area); silicate composition of ocean sediments in the Southeast and Eastern equatorial Pacific where paired ε_Nd_ vs. La/Yb* data are available (open square symbols) and where paired data are not available (light shaded area). Purple signifies the Asian dust source (dark shaded area); silicate composition of ocean sediments in the North Central Pacific where paired ε_Nd_ vs. La/Yb* data are available (open square symbols) and where paired data are not available (light shaded area). ε_Nd_ range of GP3 core at the EOT and Mesozoic/Cenozoic Andean volcanic are shown as vertical dashed arrows. See refs 24,25,27,37,59,60,63,64 for data sources. (**b**) detail from inset box shown in (a) with time-series information for *Nd and La/Yb* in our data.

A clue to the cause of the inferred northward migration in ITCZ range comes from the close association of the provenance switch with the first of the two rapid steps in benthic δ^18^O that signify accelerated cooling and onset of sustained Antarctic glaciation across the EOT (Fig. 2). This association with the initiation of a colder well-developed (unipolar) glacial climate state is unlikely to be a coincidence and suggests the operation of an inter-hemisphere teleconnection between extra-tropical cooling and low latitude rainfall consistent with recent work^40^,^41^,^42^. A full mechanistic understanding of the way in which the ITCZ may be teleconnected to extra-tropical forcing is yet to be developed but the geological record provides striking examples of shifts in the position of the thermal equator that led to pronounced hydrological reorganization in the tropics, see refs 43,44 and references therein. We estimate that, during the Late Eocene Asian dust transport season, boreal winter-spring, the ITCZ in the region ranged at least as far south as our study site, ∼0.25 ±1 °N, four degrees latitude further south than today. Asian dust supply to the equator then became blocked by a northward migration of the ITCZ in the region in step with cooling and glaciation of Antarctica. We cannot test for the full latitudinal extent of ITCZ migration using our data set alone but we can place an upper limit on its overall extent in the region because the long-term ε_Nd_ record from Site GPC3 (Fig. 1) shows no sign of a Central/South American dust signal throughout the last 40 Ma^25^, indicating that the ITCZ in the Central North Pacific remained south of ∼20 °N.

Even modest changes in ITCZ position are associated with large changes in cross-equatorial atmospheric heat transport^45^ and have important consequences for hydroclimate of the continents. Northward ITCZ migration in the eastern central equatorial Pacific today is well documented both on seasonal and inter-annual time scales associated with La Niña conditions of the El Niño Southern Oscillation and perhaps also during La Niña-like conditions suggested to coincide with persistent North American droughts such as those of the late nineteenth century^46^. It is noteworthy, therefore, that terrestrial records document a sharp Eocene-to-Oligocene transition to cooler and drier conditions on North America^18^,^19^,^47^ and in central Asia^17^,^39^,^48^,^49^. This desiccation signal is particularly well documented in the Xining Basin, northeastern Tibetan Plateau^17^,^39^ where it occurs closely associated with the observed switch in dust supply to our study site (Figs 1 and 2). This aridification event was recently attributed^16^ to a weakening of the Asian summer monsoon. That mechanism is seemingly at odds with our results unless northward ITCZ migration did not extend into the Indo-western Pacific. Regardless, different mechanisms to monsoon weakening are required to explain EOT desiccation as far north as southern Mongolia^48^ and the northwestern tip of China^49^ (Fig. 1), where the westerlies appear to have dominated moisture supply since the Eocene^31^ and a remnant Paratethys Ocean provided an upwind moisture source^16^,^39^,^50^,^51^. These observations suggest that the impact on the atmosphere of the accelerated cooling that lead to Antarctic glaciation may have extended beyond the ITCZ into the mid-latitude belt of the westerlies in the northern hemisphere^52^,^53^,^54^ (also see ref. 44 and references therein).

Our results point to the operation of extra-tropical forcing on low latitude precipitation in the central Pacific Ocean driven by climatic deterioration in the high latitudes of the southern hemisphere as Earth shifted from a largely unglaciated to a unipolar glacial climate state. The sign of our reported ITCZ migration (northward) implies that, despite the potential for Antarctic glaciation to trigger warming in parts of the Southern Ocean^14^ and the larger global land fraction north of the equator, EOT cooling was less pronounced in the northern- than in the southern-hemisphere– perhaps signifying a greater increase in planetary albedo south of the equator^55^. Further assessment is merited of existing interpretations of EOT dessication on North America and in central Asia.

## Methods

### Chronology

Our data from IODP Site U1334 in the equatorial Pacific and those from nearby ODP Site 1218 are presented on the detailed eccentricity-based orbitally tuned chronology of ref. 21. We correlated the records from Southern Ocean Site 689 to the Pacific records using the following datums as tie points: the top of each of C13n, C13r and C15n magnetochrons and the two prominent EOT δ^18^O steps (Fig. 2). To correlate the terrestrial records from the Xining Basin to our marine records we used age model-3 of ref. 39 for the Tashan section and the following datums as tie points: the top of each of C13n, C13r and C15n magnetochrons and two additional control points; (i) the last (uppermost) prominent gypsum bed (G7) and (ii) the last (uppermost) overlying regionally correlatable gypsum bed (G4), asterisked, were tied to the onset of δ^18^O step-1 in the marine records and the Eocene–Oligocene boundary, respectively (Fig. 2). The study interval, from 35.67 to 33.03 Ma, covers the entire EOT. A total of 20 samples analyzed for the interval, resulting in analytical time resolution of ca. 150 kyrs.

### Geochemistry

The inorganic silicate fraction of bulk pelagic sediments was extracted by a standard sequential treatment with a 25% acetic acid, a hot sodium citrate-sodium dithionite solution buffered with sodium bicarbonate, and a hot sodium hydroxide solution to remove carbonate components, oxides and hydroxide, and biogenic silica, respectively^29^. Sediments were digested and measured for major, trace, and rare earth elements (REEs) at the University of Southampton, National Oceanography Centre Southampton (USNOCS). Samples were digested in 15 ml PFA Savillex vials with HNO_3_-HF and heated at 130 °C overnight. After heating to dryness, the samples were re-dissolved with ∼2 ml of 6 M HCl at 130 °C overnight to remove fluoride precipitate. This process was repeated at least twice until dissolution was complete. The samples were re-dissolved with diluted HCl and then weighed. Sub-samples were taken for ICP-MS analysis. After drying down, each sub-sample was re-dissolved in a 3% HNO_3_ solution containing 10 ppb In, Re and 20 ppb Be to act as internal standards. Sample solutions were introduced into a ThemoFinnigan X-Series2 inductively coupled mass spectrometer (ICP-MS). Major, trace and REEs were quantified based on external calibration method with matrix-matched rock standard solutions (BIR1, BHVO2, JB-1a, JA-2, JGb-1, JB-3). The summed precision of the extraction procedure, sample preparation, and instrumental analysis was evaluated by analysis of three individually prepared aliquots of sample U1334B-27X-4W (11–13 cm). The total precision for each element was generally better than 6% of the measured value (2 S.D.), with the exceptions of Cr, Y and Ta which are greater than 10% of the measured values.

Sub-samples from the dissolution procedure described above were taken to obtain ∼1 μg of Nd. The Nd was isolated using AG50W-X8 cation exchange resin column to separate REEs from the matrix elements followed by an Ln-Spec resin column to separate Nd from the other REEs. The purified Nd was loaded onto an outgassed Ta side filament of a Ta-Re-Ta triple filament assembly. The ^143^Nd/^144^Nd ratios for each sample were measured using a VG Sector 54 thermal ionization mass spectrometer (TIMS) at USNOCS using a peak jumping multi dynamic routine. Isotope ratios were normalized to ^146^Nd/^144^Nd ratio of 0.7219. The long term instrument average for JNdi is ^143^Nd/^144^Nd = 0.512092 ± 15 (2 S.D., *n* = 50). Analytical reproducibility associated with column chemistry and instrumental analysis was ^143^Nd/^144^Nd ratio of 0.512283 ± 21 (2 S.D., *n* = 3) as determined by triplicate analysis of sample U1334A-27X-3W (74–76 cm). ε_Nd(0)_ values were determined by comparison to the Chondrite Uniform Reservoir (CHUR) for the present day (ε_Nd_ = ((^143^Nd/^144^Nd – 0.512638)/0.512638) × 10^4^)^56^.

The inorganic silicate fraction in our samples is dominated by eolian dust with a minor contribution from any volcanogenic material that survived the extraction steps, see ref. 26 and references therein. Any influence from volcanogenic sources in the region is readily identified because these sources are supplied sporadically and they are geochemically highly distinct from the dominant eolian fraction (see outlier with extremely high radiogenic ε_Nd_ (+1.2) and LREE-depleted La/Yb* (0.73) in Table S1). Marine barite (BaSO_4_), a common authigenic and refractory mineral phase in equatorial Pacific deep sea sediments, is present in our extracted fractions at high levels (barium contents between 0.25 ∼ 2.90 wt. %) and therefore masks the ^87^Sr/^86^Sr composition of dust through incorporation of seawater Sr and compromising Eu concentrations in the analytical process (BaO shares the same mass as Eu). Thus ^87^Sr/^86^Sr and Europium (Eu) anomalies cannot be used as dust source discriminators.

## Additional Information

**How to cite this article**: Hyeong, K. *et al.* Response of the Pacific inter-tropical convergence zone to global cooling and initiation of Antarctic glaciation across the Eocene Oligocene Transition. *Sci. Rep.* **6**, 30647; doi: 10.1038/srep30647 (2016).

## Supplementary Material

Supplementary Information
