# Supplementary material for: Response of the Pacific inter-tropical convergence zone to global cooling and initiation of Antarctic glaciation across the Eocene Oligocene Transition
Source: Sci Rep. 2016 Aug 10;6:30647. doi: 10.1038/srep30647 (PMC4979033; doi:10.1038/srep30647)
Supplement: Supplementary Information [file srep30647-s1.pdf]

Supplementary Information

Response of the Pacific inter-tropical convergence zone to global cooling and initiation of Antarctic glaciation across the Eocene Oligocene Transition

Kiseong Hyeong[1*], Junichiro Kuroda[2], Inah Seo[1,3], Paul A. Wilson[4*]

1. Korea Institute of Ocean Science and Technology, Ansan, South Korea (kshyeong@kiost.ac.kr)
2. Japan Agency for Marine-Earth Science and Technology, Yokosuka, Japan (kurodaj@jamstec.go.jp)
3. School of Earth and Environmental Sciences, Seoul National University, Seoul, South Korea (inahseo@snu.ac.kr)
4. National Oceanography Center Southampton, University of Southampton, Waterfront Campus, Southampton, SO14 3ZH, UK (paul.wilson@noc.soton.ac.uk)

*Corresponding authors: PAW(paul.wilson@noc.soton.ac.uk) and KH(kshyeong@kiost.ac.kr)

Table S1. Isotopic and chemical compositions of study samples.

Sample ID[a]	Depth	Age[b]	$^{87}Sr/^{86}Sr$		$^{143}Nd/^{144}Nd$		ε_{Nd}[e]	Li	Na	Mg	Al	K	Ca	Ba	Sc	Ti	V	Cr	Mn	Fe	Co	Ni	Cu	Zn	Rb
	(m)	(Ma)		SE		SE		ppm	%	%	%	%	%	%	ppm	ppm	ppm	ppm	%	%	ppm	ppm	ppm	ppm	ppm
U1334A-26-3-75	287.0	33.03	0.70831	7	0.512272	7	-7.1	80.5	1.03	1.58	3.25	1.06	0.77	2.95	20.5	0.20	41.5	25.7	0.24	5.05	3.65	151	386	186	53.5
U1334A-26-4-76	288.5	33.15	0.70829	8	0.512349	8	-5.6	80.9	1.02	1.45	2.44	0.78	0.67	1.65	16.8	0.15	30.3	23.8	0.26	4.42	2.77	156	358	152	38.4
U1334A-26-5-76	290.0	33.26	0.70866	9	0.512294	7	-6.7	69.2	1.25	1.17	3.14	0.92	0.84	0.24	13.3	0.20	39.2	25.1	0.22	3.42	3.55	120	221	130	43.5
U1334A-26-6-76	291.5	33.39	0.70838	8	0.512336	7	-5.9	124	2.04	2.69	4.57	1.37	0.96	0.86	27.8	0.27	54.2	29.6	0.43	9.55	4.79	252	593	300	67.4
U1334B-26-1-117	291.7	33.40	0.70828	7				128	1.79	2.32	3.58	1.02	0.80	1.43	26.5	0.23	41.4	25.6	0.42	8.12	3.76	237	566	260	52.7
U1334B-26-2-76	292.8	33.48	0.70819	9	0.512349	7	-5.6	136	1.73	2.53	4.15	1.18	0.99	1.86	26.6	0.25	50.7	29.4	0.38	9.00	4.62	255	630	288	61.4
U1334B-26-4-74	295.8	33.68	0.70814	8	0.512342	9	-5.8							0.00											
U1334B-26-5-74	297.3	33.77	0.70830	8	0.512295	21	-6.7	116	1.62	2.46	4.96	1.57	0.87	2.05	30.0	0.31	58.8	27.0	0.35	9.07	5.11	202	569	285	79.9
U1334A-27-3-74	298.0	33.83	0.70817	8	0.512293	7	-6.9	71.4	1.41	1.60	4.74	1.64	0.91	2.22	22.3	0.26	52.4	24.6	0.16	5.51	4.30	105	327	190	81.8
U1334A-27-4-74	299.0	33.95	0.70822	7	0.512355	6	-5.5	128	1.39	2.85	4.61	1.52	0.93	2.29	33.0	0.33	50.5	27.2	0.35	10.1	4.77	213	579	261	77.0
U1334A-27-5-74	300.0	34.15	0.70808	8	0.512206	9	-8.4	68.8	1.58	1.92	3.62	1.14	0.77	2.46	24.1	0.22	40.4	18.6	0.21	7.58	3.39	114	393	225	61.5
U1334C-28-2-74	300.6	34.25	0.70815	7	0.512181	8	-8.9	51.5	1.11	1.43	3.32	1.05	0.74	1.71	19.7	0.19	39.7	19.2	0.12	5.34	3.22	65.3	232	171	55.3
U1334A-27-6-58	301.4	34.41	0.70811	7	0.512189	8	-8.8	88.7	1.67	2.53	3.05	1.06	0.71	2.22	25.9	0.21	34.3	19.8	0.21	9.81	2.92	136	565	246	59.5
U1334C-28-3-74	302.1	34.53												1.55	8.65										
U1334B-27-1-74[c]	302.7	34.66	0.70823	8	0.512161	139	-9.3	53.2	1.23	1.32	3.32	1.32	0.70	1.61	16.8	0.15	33.7	16.2	0.08	4.65	2.82	50.7	256	156	76.6
U1334B-27-1-76[d]	302.7	34.66	0.70842	7	0.512701	21	1.2	63.1	1.53	1.47	4.06	1.94	0.77	1.17	16.1	0.17	33.8	17.6	0.07	5.40	3.17	53.0	264	180	105
U1334B-27-2-73	304.2	34.92												1.72	19.8										
U1334B-27-2-76	304.2	34.93	0.70828	7				59.5	0.85	1.49	2.97	1.00	0.74	1.60	23.5	0.18	33.8	18.6	0.07	5.06	3.06	54.3	223	146	48.3
U1334B-27-3-72	305.7	35.11	0.70827	8	0.512209	7	-8.4	72.1	1.54	2.23	3.89	1.39	0.73	1.56	24.6	0.22	44.3	22.7	0.12	8.50	3.50	70.6	424	256	70.2
U1334B-27-3-76	305.7	35.12	0.70842	7	0.512235	6	-7.9	78.2	1.36	2.19	3.53	1.29	0.76	1.18	23.0	0.22	40.7	22.1	0.12	8.41	3.72	61.9	417	232	63.8
U1334B-27-4-11	306.5	35.29	0.70825	8	0.512238	5	-7.8	93.8	1.66	3.06	3.34	1.28	0.75	1.86	23.4	0.24	37.4	24.1	0.16	11.9	3.73	70.3	552	270	67.8
U1334A-28-1-74	306.9	35.32	0.70821	8	0.512202	7	-8.5	94.3	1.57	2.84	2.82	0.93	0.75	1.83	27.4	0.21	30.3	19.8	0.13	10.6	2.90	82.0	600	207	52.6
U1334A-28-2-74	308.4	35.50	0.70820	8	0.512239	5	-7.8	97.8	1.63	3.11	3.63	1.37	0.84	1.70	27.2	0.23	38.6	22.2	0.14	11.2	3.58	67.2	561	241	67.9
U1334A-28-3-74	309.9	35.67	0.70817	6	0.512194	6	-8.7	57.6	1.25	1.69	2.91	1.02	0.66	1.48	17.0	0.15	32.2	16.9	0.13	6.46	2.75	41.0	289	195	53.0

[a]site-hole-core-section-depth(top, cm), [b]age model is from Westerhold et al. (2014) [1], [c]this sample has a large analytical error in the determined $^{143}Nd/^{144}Nd$ and is excluded from discussion. [d]this sample has high radiogenic ε_{Nd} (+1.2) and LREE-depleted La/Yb* (0.73), indicative of episodic input of volcanogenic materials and is excluded from discussion, [e]The $\varepsilon_{Nd(0)}$ values were determined by comparison to the Chondrite Uniform Reservoir (CHUR) for the present day, where $(^{143}Nd/^{144}Nd)_{CHUR} = 0.512638$ [2].

Table S1. (continued)

Sample ID	Sr	Y	Zr	Nb	La	Ce	Pr	Nd	Sm	Eu	Eu[f]	Gd	Tb	Dy	Ho	Er	Tm	Yb	Lu	Hf	Pb	Th	U	La/Yb*[g]
	ppm	ppm	ppm	ppm	ppm	ppm	ppm	ppm	ppm	ppm	ppm	ppm	ppm	ppm	ppm	ppm	ppm	ppm	ppm	ppm	ppm	ppm	ppm	
U1334A-26-3-75	694	5.70	193	5.53	7.43	14.4	1.64	5.93	1.10	0.25	0.79	1.10	0.14	0.88	0.19	0.61	0.10	0.66	0.10	3.88	73.7	1.78	0.57	0.83
U1334A-26-4-76	527	5.16	155	3.95	5.82	11.2	1.28	4.67	0.90	0.21	0.52	0.79	0.12	0.76	0.17	0.56	0.08	0.59	0.09	3.18	49.1	1.26	0.39	0.72
U1334A-26-5-76	261	4.76	162	5.02	5.96	11.4	1.30	4.77	0.90	0.25	0.31	0.67	0.12	0.69	0.16	0.51	0.08	0.52	0.08	3.27	44.7	1.55	0.51	0.85
U1334A-26-6-76	478	6.19	237	6.43	6.91	13.5	1.57	5.73	1.08	0.30	0.47	0.91	0.15	0.93	0.21	0.65	0.10	0.71	0.11	4.44	42.7	1.79	0.58	0.72
U1334B-26-1-117	571	4.85	249	5.21	5.75	10.7	1.24	4.52	0.86	0.23	0.50	0.73	0.12	0.71	0.17	0.54	0.08	0.56	0.09	4.76	###	1.33	0.44	0.76
U1334B-26-2-76	761	5.52	244	5.84	6.12	11.8	1.38	5.07	0.99	0.26	0.59	0.90	0.13	0.81	0.18	0.60	0.09	0.63	0.10	4.58	19.6	1.68	0.57	0.72
U1334B-26-4-74																								
U1334B-26-5-74	714	7.38	242	7.40	9.20	16.9	2.01	7.15	1.34	0.35	0.72	1.29	0.18	1.17	0.25	0.79	0.12	0.86	0.14	4.78	38.2	2.23	0.72	0.79
U1334A-27-3-74	676	9.86	176	8.20	11.4	22.0	2.47	8.63	1.66	0.39	0.77	1.54	0.23	1.43	0.32	1.01	0.16	1.13	0.18	3.80	37.9	3.83	1.10	0.74
U1334A-27-4-74	920	7.33	275	7.95	8.84	16.7	1.93	6.90	1.31	0.32	0.73	1.28	0.18	1.13	0.25	0.77	0.12	0.83	0.13	5.37	72.4	2.42	0.75	0.78
U1334A-27-5-74	632	4.61	150	6.00	6.26	11.6	1.35	4.89	0.89	0.25	0.67	0.85	0.11	0.69	0.16	0.50	0.08	0.53	0.09	3.05	37.0	1.50	0.50	0.87
U1334C-28-2-74	489	4.59	132	5.05	6.80	12.6	1.47	5.22	0.91	0.26	0.57	0.78	0.11	0.70	0.16	0.50	0.08	0.53	0.09	2.52	36.3	1.46	0.50	0.94
U1334A-27-6-58	684	3.80	148	5.59	5.64	10.4	1.20	4.22	0.77	0.21	0.61	0.62	0.09	0.57	0.14	0.43	0.06	0.44	0.07	2.89	66.7	1.30	0.44	0.95
U1334C-28-3-74		2.81			4.14	7.74	0.89	3.17	0.58	0.16	0.45	0.47	0.07	0.39	0.10	0.32	0.05	0.31	0.05		20.7			1.00
U1334B-27-1-74	504	9.48	131	7.16	9.78	19.2	2.16	7.46	1.47	0.23	0.52	1.31	0.21	1.37	0.30	0.94	0.15	1.08	0.17	2.82	28.2	4.33	1.38	0.67
U1334B-27-1-76	436	13.1	154	9.67	11.8	24.0	2.61	8.92	1.79	0.22	0.45	1.60	0.28	1.81	0.41	1.29	0.21	1.19	0.24	3.49	31.4	7.80	2.41	0.74
U1334B-27-2-73		5.10			7.90	15.0	1.73	6.22	1.11	0.31	0.64	0.92	0.13	0.79	0.18	0.58	0.09	0.59	0.10		40.0			0.98
U1334B-27-2-76	406	5.16	144	4.55	7.71	14.5	1.68	5.98	1.08	0.29	0.58	0.89	0.13	0.78	0.18	0.56	0.09	0.59	0.09	2.83	24.7	1.32	0.43	0.97
U1334B-27-3-72	502	4.37	182	5.74	6.40	12.0	1.40	5.01	0.93	0.24	0.53	0.73	0.11	0.66	0.16	0.49	0.07	0.52	0.08	3.38	28.2	1.46	0.50	0.90
U1334B-27-3-76	423	4.38	186	5.36	6.23	11.6	1.35	4.84	0.87	0.24	0.47	0.70	0.11	0.65	0.15	0.49	0.07	0.51	0.08	3.41	46.5	1.42	0.49	0.90
U1334B-27-4-11	605	3.80	218	5.98	5.37	10.0	1.15	4.07	0.73	0.20	0.54	0.61	0.09	0.55	0.13	0.42	0.06	0.43	0.07	3.87	49.6	1.36	0.46	0.92
U1334A-28-1-74	633	3.47	197	5.51	4.96	9.13	1.04	3.70	0.66	0.20	0.53	0.48	0.08	0.50	0.12	0.39	0.06	0.39	0.06	3.77	61.3	1.14	0.37	0.94
U1334A-28-2-74	629	4.34	218	6.27	5.89	10.8	1.25	4.50	0.83	0.24	0.54	0.70	0.10	0.64	0.15	0.49	0.07	0.51	0.08	4.20	40.8	1.40	0.47	0.86
U1334A-28-3-74	446	3.64	138	4.70	5.07	9.09	1.08	3.80	0.68	0.19	0.44	0.55	0.08	0.51	0.12	0.41	0.06	0.40	0.07	2.67	18.7	1.15	0.39	0.93

[f]uncorrected for isobaric interferences (see method section for details), [g]$(La/Yb)_{SAMPLE}/(La/Yb)_{PAAS,}$ PAAS(Post-Archean Australian Average Shale [3])-normalized Lanthanium (La) to Ytterbium (Yb) ratio

References cited

1 Westerhold, T. *et al.* Orbitally tuned timescale and astronomical forcing in the middle Eocene to early Oligocene. *Climate of the Past* **10**, 955-973, doi:10.5194/cp-10-955-2014 (2014).

2 Jacobsen, S. B. & Wasserburg, G. J. Sm-Nd isotopic evolution of chondrites. *Earth and Planetary Science Letters* **50**, 139-155, doi:http://dx.doi.org/10.1016/0012-821X(80)90125-9 (1980).

3 Taylor, S. R. & McLennan, S. M. *The continental crust: Its composition and evolution*. (1985).
